# Long-term major adverse cardiovascular events following myocardial injury after non-cardiac surgery: meta-analysis

**DOI:** 10.1093/bjsopen/zrad021

**Published:** 2023-04-25

**Authors:** Scarlett S Strickland, Ella M Quintela, Matthew J Wilson, Matthew J Lee

**Affiliations:** Academic Directorate of General Surgery, Sheffield Teaching Hospitals, Sheffield, UK; Department of Anaesthesia, Sheffield Teaching Hospitals, Sheffield, UK; Centre for Urgent and Emergency Care Research, School of Health and Related Research, University of Sheffield, Sheffield, UK; Department of Anaesthesia, Sheffield Teaching Hospitals, Sheffield, UK; Centre for Urgent and Emergency Care Research, School of Health and Related Research, University of Sheffield, Sheffield, UK; Academic Directorate of General Surgery, Sheffield Teaching Hospitals, Sheffield, UK; Department of Oncology and Metabolism, The Medical School, University of Sheffield, Sheffield, UK

## Abstract

**Background:**

Myocardial injury after non-cardiac surgery is diagnosed following asymptomatic troponin elevation in the perioperative interval. Myocardial injury after non-cardiac surgery is associated with high mortality rates and significant rates of major adverse cardiac events within the first 30 days following surgery. However, less is known regarding its impact on mortality and morbidity beyond this time. This systematic review and meta-analysis aimed to establish the rates of long-term morbidity and mortality associated with myocardial injury after non-cardiac surgery.

**Methods:**

MEDLINE, Embase and Cochrane CENTRAL were searched, and abstracts screened by two reviewers. Observational studies and control arms of trials, reporting mortality and cardiovascular outcomes beyond 30 days in adult patients diagnosed with myocardial injury after non-cardiac surgery, were included. Risk of bias was assessed using the Quality in Prognostic Studies tool. A random-effects model was used for the meta-analysis of outcome subgroups.

**Results:**

Searches identified 40 studies. The meta-analysis of 37 cohort studies found a rate of major adverse cardiac events-associated myocardial injury after non-cardiac surgery of 21 per cent and mortality following myocardial injury after non-cardiac surgery was 25 per cent at 1-year follow-up. A non-linear increase in mortality rate was observed up to 1 year after surgery. Major adverse cardiac event rates were also lower in elective surgery compared with a subgroup including emergency cases. The analysis demonstrated a wide variety of accepted myocardial injury after non-cardiac surgery and major adverse cardiac events diagnostic criteria within the included studies.

**Conclusion:**

A diagnosis of myocardial injury after non-cardiac surgery is associated with high rates of poor cardiovascular outcomes up to 1 year after surgery. Work is needed to standardize diagnostic criteria and reporting of myocardial injury after non-cardiac surgery-related outcomes.

**Registration:**

This review was prospectively registered with PROSPERO in October 2021 (CRD42021283995).

## Teaser

Whilst it is acknowledged that myocardial injury following non-cardiac surgery is associated with poor cardiovascular outcomes within the immediate perioperative interval, little is known about the long-term sequelae of myocardial injury following non-cardiac surgery. This systematic review and meta-analysis has demonstrated that myocardial injury following non-cardiac surgery is associated with high rates of mortality and major adverse cardiac events up to 1 year following non-cardiac surgery.

## Introduction

Cardiac complications remain a leading cause of postoperative morbidity and mortality^1,2^. The occurrence of cardiac complications following coronary intervention is well described by the 4th Universal Definition of Myocardial Infarction^3^. However, there is increasing recognition of myocardial injury following non-cardiac surgery (MINS)^4^. A diagnosis of MINS is made through the presence of elevated cardiac troponin levels, thought to be ischaemic in nature, without associated ischaemic features (for example chest pain or ECG changes), which occur within 30 days of surgery^2,3,5–7^.

MINS is thought to result from an imbalance in myocardial oxygen supply and demand arising during an acute interval of illness^3^. The exact mechanism as to how this occurs remains largely unknown. Recent studies suggest that acute postoperative endothelial dysfunction has a role to play^8^, in particular, impaired endothelial nitrous oxide production^9^. Underlying vagal dysfunction, leading to an inability to adapt to the physiological stresses of surgery, has also been suggested as a potential cause of MINS^10,11^.

MINS may occur following at least 8 per cent of elective procedures^2,12^ and up to 25 per cent of emergency surgery cases^13^. It is associated with increased mortality within 30 days following surgery^7^ as well as longer length of inpatient stay^2,14^. Furthermore, evidence has emerged to suggest a link between MINS and the occurrence of major adverse cardiac events (MACE). This appears to persist despite controlling for other confounders such as baseline cardiac risk^15^. Although there is no universally accepted definition of MACE^16^, components frequently reported in studies include myocardial infarction, non-haemorrhagic stroke, arrhythmia, heart failure, peripheral arterial thrombosis, cardiac arrest and amputation^2,15,17^.

The incidence of MACE following MINS is reported frequently in the first 30 days; however, less is known about the long-term sequelae of MINS^2^. A systematic review in 2020 has highlighted that beyond 1 year after surgery, mortality rates of patients with MINS remain statistically higher than those without the diagnosis^18^. Therefore, this systematic review and meta-analysis aims to establish long-term morbidity and mortality rates, in the context of MACE outcomes, in patients diagnosed with MINS.

## Methods

This systematic review was reported in line with the PRISMA guidelines^19^ and conducted with reference to the Cochrane Handbook and Meta-analyses Of Observational Studies in Epidemiology (MOOSE) guidelines^20,21^.

A search strategy (*Appendix S1*) was devised by S.S. and M.J.L. with reference to previous reviews regarding MINS^15,18,22^. Searches of the following electronic databases were conducted in October 2021 via MEDLINE (via OvidSP), Embase (via OvidSP) and Cochrane CENTRAL (1974–2021 with no other limits applied).

All search results were exported onto a software database, Rayyan^23^, and duplicates were then removed. Abstracts were screened for inclusion by two reviewers (S.S. and E.Q.) and conflicts resolved by a third (M.J.L.). Full texts were retrieved for included abstracts. Full texts were screened against eligibility criteria and extracted onto a predesigned data extraction sheet created in Microsoft Excel (Microsoft Corporation, Redmond, VA, USA) by two authors (S.S. and E.Q.). This form included study descriptors, type of operation, definition of MINS and MACE, study population with MINS, end time of study and included outcomes at day 30, 60, 90 and 1 year after surgery.

### Eligibility criteria

Studies reporting outcomes 30 days or more following surgery in patients who developed myocardial injury after MINS were included. For this study, MINS was defined as any value of troponin reaching a predefined threshold without associated ECG changes.

Only studies involving adult patients (aged 18 years and above) were included. Cohort studies and control arms of interventional studies treating MINS in any non-cardiac surgical setting were considered.

Studies reporting on paediatric patients (aged under 18 years), those who underwent cardiac surgery and patients who were not diagnosed with myocardial injury were excluded. Studies that did not stipulate their diagnostic criteria for MINS were also excluded. Case reports, diagnostic studies and studies reporting on intervention arms of RCTs were also excluded.

### Primary outcome

The primary outcome was mortality and any cardiovascular complication within the MACE definition which was defined by the original study. These definitions were also recorded. Where available, aggregate MACE rates and components of MACE were extracted to address heterogeneity between studies that may have variations in reported outcomes. Accepted MACE components include myocardial infarction, non-haemorrhagic stroke, arrhythmia, heart failure, peripheral arterial thrombosis, cardiac arrest, amputation and death. Where available, event rates of mortality and MACE components were documented at 30 days, 60 days, 90 days, 6 months and 1 year.

### Qualitative synthesis

For each included study, the definitions used to diagnose myocardial injury and MACE were extracted. Data on event rates were extracted where meta-analysis was not possible.

### Statistical analysis and planned subgroup analyses

From baseline data, subgroups were formed based on type of surgery by specialty and acuity of surgery. Planned subgroup analyses were comparison of mortality and MACE events between subgroups. Where three or more studies reported an outcome of interest, the Mantel–Haenszel random-effects approach was used to meta-analyse the proportions with subgroups according to acuity of surgery or type of surgery as appropriate.

Summary event rates were calculated for the whole population and each subgroup, with 95 per cent confidence intervals. If subgroups consisted of studies with differing follow-up intervals, for example when comparing between different types of surgery and acuity, event rates were calculated to events per day. Analyses were conducted using R statistics and the Meta package^24^.

### Bias assessment

Two reviewers (S.S. and E.Q.) assessed each included study for risk of bias using the validated Quality in Prognostic Studies tool (QUIPS)^25^. QUIPS was selected as the studies used MINS as a risk factor for cardiovascular outcomes. The QUIPS tool was used to assess risk of bias for each included study. Risk of bias was assessed across six domains: study participation, study attrition, prognostic factor, outcome measure, confounding and statistical analysis. Each domain included subheadings to facilitate and standardize the interrater bias assessment^26^. Disagreements in bias scores were resolved through discussion. No studies were excluded from the analysis based on the results of bias assessment.

## Results

**Fig. 1:**
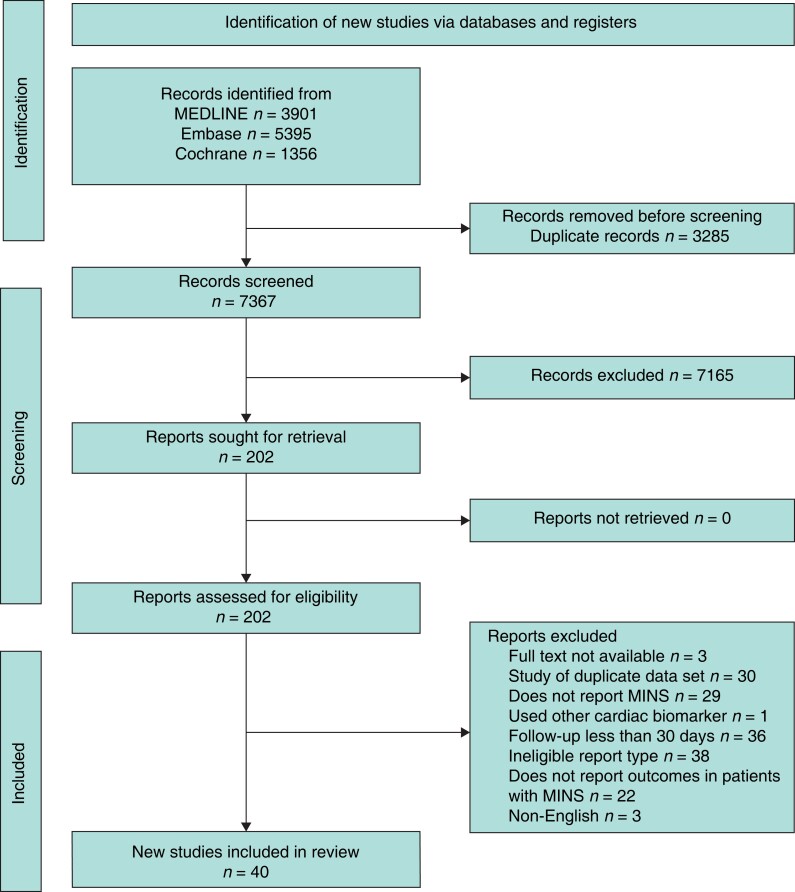
**PRISMA flow diagram of study selection** ^27^

A total of 10 652 studies were initially identified from searches. Following the removal of duplicates, 7367 abstracts were screened. Screening excluded 7165 abstracts leading to 202 full texts undergoing assessment for eligibility. Based on predefined inclusion and exclusion criteria, 162 studies were excluded resulting in 40 studies being included in the final analysis (*Fig. 1*).

MINS, myocardial injury after non-cardiac surgery.

### Study characteristics

A summary of study characteristics is included in *Table 1*. The majority of studies included all types of non-cardiac surgery in their eligibility criteria^2,15,22,30,33–35,37,38,40,43,44,46–49,51,56,58,60–62,65^. Seventeen studies focused on specific surgical subspecialties including vascular^28,39,41,42,45,52–54^, orthopaedic^29,31,32,50,55,59,64^, urology^36,63^ and thoracic surgery^36,63^. Regarding surgical acuity, only two studies focused on emergency non-cardiac surgery^31,32^, whilst nine studies specifically included only patients undergoing elective surgery^28,35–38,52,53,58,63^.

**Table 1:** Summary of included studies: study design, surgery type and follow-up interval

Author (year)	Design	Type of surgery	Emergency or elective	Follow-up interval (days)
Ali *et al.* 2008^28^	Cohort	Vascular	Elective	571
Auroy *et al.* 2008^29^	Cohort	Orthopaedic	Mix	365
Beattie *et al.* 2012^22^	Cohort	Non-cardiac	Mix	30
Botto *et al.* 2014*^2^*	Cohort	Non-cardiac	Mix	30
Canbolat *et al.* 2014^30^	Cohort	Non-cardiac	Not specified	365
Chong *et al.* 2009^31^	Cohort	Orthopaedic	Emergency	365
Chong *et al.* 2012^32^	Control arm of RCT	Orthopaedic	Emergency	365
Devereaux *et al.* 2018^15^	Control arm of RCT	Non-cardiac	Not specified	730
Filipovic *et al.* 2003^33^	Cohort	Non-cardiac	Not specified	365
Genc Moralar *et al.* 2021^34^	Cohort	Non-cardiac	Mix	30
George *et al.* 2018^35^	Cohort	Non-cardiac	Elective	30
Gonzalez- Tallada *et al.* 2020^36^	Cohort	Thoracic	Elective	30
Gouda *et al.* 2021^37^	Cohort	Non-cardiac	Elective	180
Hallqvist *et al.* 2016^38^	Cohort	Non-cardiac	Elective	180
Hobbs *et al.* 2005^39^	Cohort	Vascular	Not specified	180
Jackson *et al.* 2018^40^	Cohort	Non-cardiac	Mix	180
Kertai *et al.* 2004^41^	Cohort	Vascular	Not specified	1570
Kim *et al.* 2002 ^42^	Cohort	Vascular	Not specified	180
Kim *et al.* 2016^43^	Cohort	Non-cardiac	Not specified	2555
Kim *et al.* 2021^44^	Cohort	Non-cardiac	Mix	365
Kisten and Biccard 2016^45^	Cohort	Vascular	Mix	Not specified
Kler *et al.* 2021^46^	Cohort	Non-cardiac	Not specified	90
Lee *et al.* 2019^47^	Cohort	Non-cardiac	Mix	760
Lee *et al.* 2020^48^	Cohort	Non-cardiac	Mix	30
Mol *et al.* 2019^49^	Cohort	Non-cardiac	Mix	365
Oberweis *et al.* 2015^50^	Cohort	Orthopaedic	Mix	1095
Oscarsson *et al.* 2004^51^	Cohort	Non-cardiac	Mix	365
Pereira-Macedo *et al.* 2019^52^	Cohort	Vascular	Elective	1570
Pereira-Macedo *et al.* 2020^53^	Cohort	Vascular	Elective	1550
Reed *et al.* 2017^54^	Cohort	Vascular	Not specified	1825
Rostagno *et al.* 2019^55^	Cohort	Orthopaedic	Not specified	365
Sazgary *et al.* 2020^56^	Cohort	Non-cardiac	Mix	365
Szczeklik *et al.* 2018^57^	Cohort	Vascular	Not specified	365
Toda *et al.* 2020^58^	Control arm of RCT	Non-cardiac	Elective	30
Vacheron *et al.* 2021^59^	Cohort	Orthopaedic	Not specified	365
Van Waes *et al.* 2016^60^	Cohort	Non-cardiac	Mix	365
Van Waes *et al.* 2017^61^	Cohort	Non-cardiac	Mix	365
Vasireddi *et al.* 2021^62^	Cohort	Non-cardiac	Not specified	365
Yu *et al.* 2020^63^	Cohort	Urology	Elective	365
Yuan *et al.* 2019^64^	Cohort	Orthopaedic	Mix	365

The length of follow-up for all included studies was recorded in days. Follow-up intervals are summarized in *Table 1* and ranged from 30 to 2555 days, with a median follow-up interval of 365 days. Due to the variety in follow-up intervals, event rates were converted to a per-day rate to allow comparison.

Outcomes reported are summarized in *Table S1*. Thirty five of the 38 included cohort studies reported mortality^2,22,28–31,33–47,49,51–55,57,59–64,66^. For MACE rates, myocardial infarction was the most frequently reported outcome (16)^28,29,31,37,48,50–53,56,57,59–61,64,65^, followed by heart failure (9)^2,28,31,37,48,53,56,64,65^ and arrhythmia (5)^31,36,48,56,65^. All three control arms from randomized controlled studies also reported rates of death or mortality, MACE and myocardial infarction^15,32,58^. None of the included studies reported outcomes for cardiac arrest or amputation.

### Definitions

A summary of the diagnostic criteria used by included studies for MINS is shown in *Table S2*.

Of the included 40 studies, 19 used high-sensitivity troponins to diagnose MINS^31,32,34–36,38,40,46–49,52,53,56–59,62,64^. Twenty-five studies used troponin I^22,28–33,35,36,39,40,42–45,48,50,52,53,59–64^, whilst 12 used troponin T assays^2,34,38,41,46,47,49,51,54,56–58^. Variation was seen between studies regarding sampling frequency, ranging from immediately after surgery to anytime within 30 days of surgery. Eight studies measured troponin preoperatively as well as postoperatively^31–33,51,56–58,67^. Definitions of MACE used by studies for reporting outcomes are summarized in *Table S3*.

### Meta-analysis

Thirty-seven studies were included in the meta-analysis of cohort studies^2,22,28–31,33–46,50–57,60–64^. *Table 2* summarizes event rates by meta-analysis by follow-up interval. Zero studies reporting outcome rates at 60 and 90 days led to these time points being excluded. The numbers of peripheral arterial thrombosis and amputation events were insufficient to permit meta-analysis. *Table 3* summarizes the outcome of meta-analysis of cohort studies for mortality and components of MACE by type of surgery. *Table 4* provides a summary of the meta-analysis of cohort studies for mortality and components of MACE by surgical acuity.

**Table 2:** Summary table of meta-analysis outcomes for cohort studies by follow-up interval

Event	Follow-up interval					
	Per cent with event at 1 month (95% c.i.)	Per cent with event at 6 months (95% c.i.)	Per cent with event at 1 year (95% c.i.)	Per cent with event at 2 years (95% c.i.)	Per cent with event beyond 2 years (95% c.i.)	Overall event rate pooled result (95% c.i.)
Death	9 (3 to 24)	8 (6 to 12)	25 (21 to 30)	*	38 (22 to 57)	22 (17 to 28)
MACE	*	*	21 (12 to 34)	*	*	25 (14 to 39)
Arrhythmia	*	*	*	*	*	12 (6 to 22)
Heart failure	*	*	*	*	*	11 (8 to 16)
Non-haemorrhagic stroke	*	*	*	*	*	2 (2 to 3)
Myocardial infarction	*	*	12 (8 to 18)	*	28 (7 to 68)	13 (8 to 20)

* Inadequate number of studies for meta-analysis. MACE , major adverse cardiac events.

**Table 3:** Summary table of meta-analysis of cohort studies for mortality and components of MACE by type of surgery (events per day)

Type of surgery	Death	Overall MACE	Stroke	Arrhythmia	MI	CCF
	Events/day (95% c.i.)					
Non-cardiac	0.23 (0.07–0.71)	0.2 (0.08–0.5)	*	*	0.19 (0.04–0.84)	*
Vascular	0.02 (0.01–0.05)	0.02 (0.01–0.06)	*	*	0.01 (0.00–0.03)	*
Orthopaedic	0.046 (0.01–0.05)	*	*	*	0.03 (0.01–0.05)	*
Thoracic	*	*	*	*	*	*
Urology	*	*	*	*	*	*
Pooled	0.08 (0.04–0.18) *P* <0.01	0.05 (0.02–0.13) *P* <0.01	0.01 (0.00–0.27) *P* <0.01	*	0.05 (0.02–0.13) *P* = 0.01	*

* Inadequate number of studies for meta-analysis. MACE, major adverse cardiac events; MI, myocardial infarction; CCF, congestive cardiac failure.

**Table 4:** Summary table of meta-analysis of cohort studies for mortality and components of MACE by surgical acuity (events per day)

Surgical acuity	Death	Overall MACE	Stroke	Arrhythmia	MI	CCF
	Events/day (95% c.i.)					
Mix	0.29 (0.06–1.35)	0.13 (0.03–0.68)	*	*	0.05 (0.02–0.16)	0.04 (0.01–0.27)
Not specified	0.05 (0.03–0.09)	*	*	*	0.04 (0.02–0.10)	*
Elective	0.03 (0.01–0.13)	0.02 (0.01–0.07)	*	*	0.02 (0.00–0.59)	0.03 (0.00–1.65)
Emergency	*	*	*	*	*	*
Pooled	0.08 (0.04–0.18) *P* = 0.14	0.05 (0.02–0.13) *P* < 0.01	0.01 (0.00–0.27) *P* < 0.01	0.14 (0.03–0.59) *P* < 0.01	0.04 (0.02–0.09) *P* = 0.70	0.04 (0.01–0.17) *P* = 0.71

* Inadequate number of studies for meta-analysis of subgroup. MACE, major adverse cardiac events; MI, myocardial infarction; CCF, congestive cardiac failure.

Three control arms^6,32,58^ were included for analysis with follow-up periods ranging from 30^58^ to 730^15^ days. Event rate analysis was limited to death, MACE and myocardial infarction due to availability of reported outcomes.

### Mortality

**Fig. 2:**
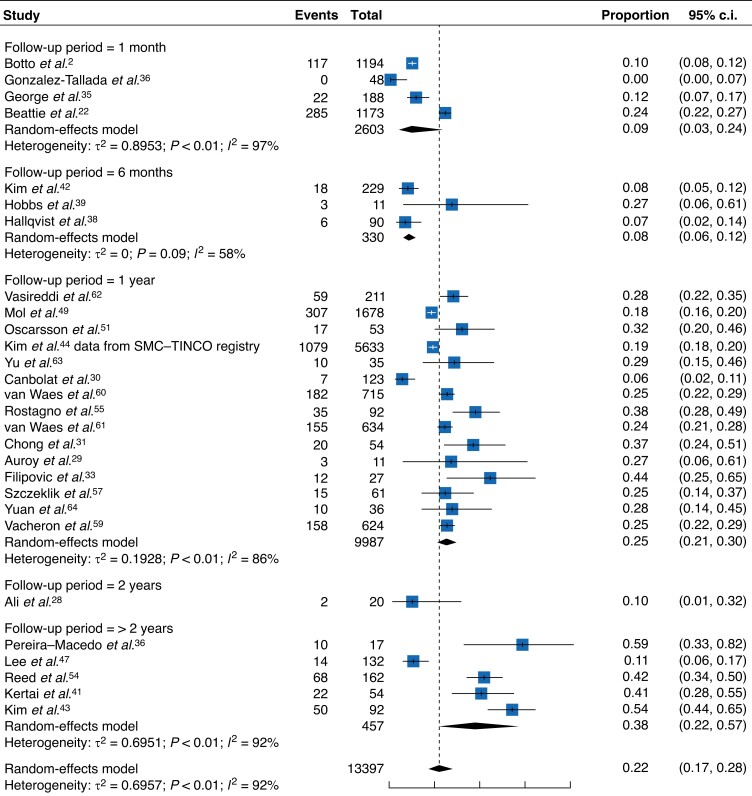
Meta-analysis of death reporting in cohort studies with subgroups of follow-up interval

The meta-analysis of eligible cohort studies demonstrated that the mortality rate associated with MINS sharply increases from 8 per cent (95 per cent c.i. 6–12 per cent) at 6 months^38,39,42^ to 25 per cent at 1 year^29–31,33,44,49,51,55,57,59–63–64^ (95 per cent c.i. 21–30 per cent) (*Fig. 2*). This increase plateaus towards 38 per cent beyond 2 years (95 per cent c.i. 22–57 per cent)^41,43,47,53,54^ (*Table 2*).

SMC-TINCO registry, Samsung Medical Center Troponin in Non cardiac Operation registry.

**Fig. 3:**
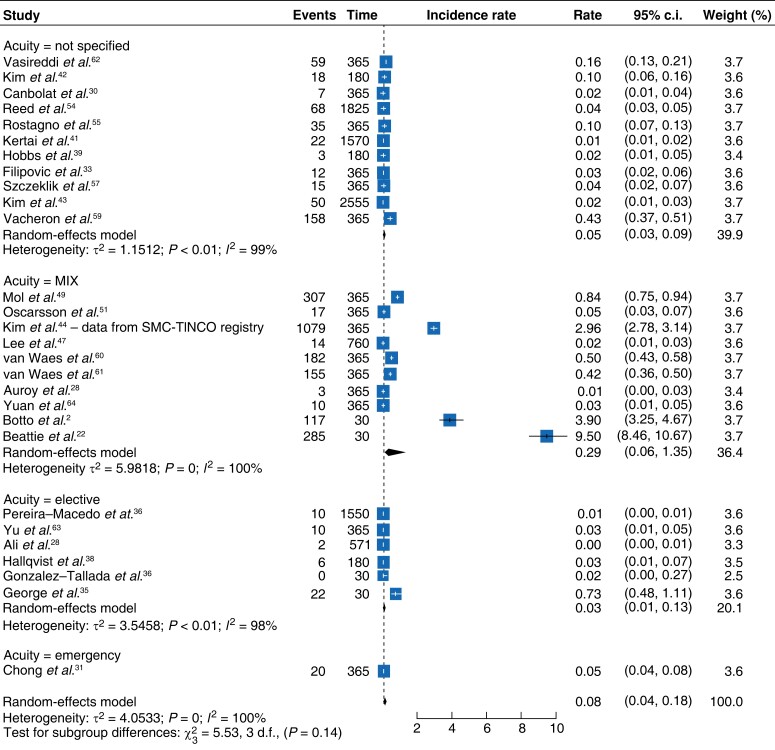
Forest plot showing meta-analysis of death rates per day in cohort studies according to acuity

**Fig. 4:**
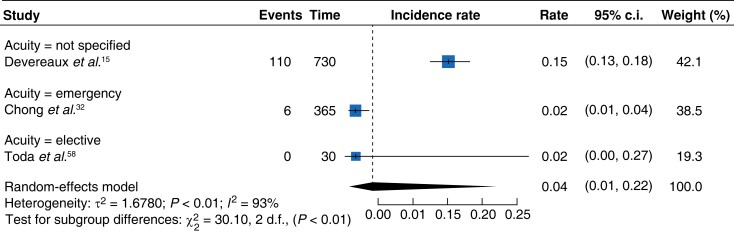
Forest plot showing meta-analysis of control arms demonstrating deaths per day

The pooled rate of MINS-associated mortality was 0.08 deaths per day (95 per cent c.i. 0.04–0.18) (*Fig. 3*)^2,22,28–31,33,35,36,38,39,41–44,47,49,51,53–55,57,59–64^. This is similar to rates observed from the meta-analysis of control arm studies. These demonstrated a pooled mortality rate of 0.04 (95 per cent c.i. 0.01–0.22) (*Fig. 4*)^15,32,58^.

MIX, combined emergency and elective.

SMC-TINCO registry, Samsung Medical Center Troponin in Non cardiac Operation registry.

Subgroup analysis by acuity (*Fig. 3*) demonstrated that elective surgery had a death rate per day of 0.03 (95 per cent c.i. 0.01–0.13)^28,35,36,38,53,63^. Studies including a mix of emergency and elective cases had a death rate of 0.29 (95 per cent c.i. 0.06–1.35)^2,22,29,44,49,51,60,61,64^.

With regard to surgical specialties, vascular surgery was associated with a mortality rate of 0.02 (95 per cent c.i. 0.01–0.05) per day^28,39,41,42,45,57^, whilst the mortality rate for orthopaedic patients was 0.06 (95 per cent c.i. 0.02–0.21) per day^29,31,55,59,64^ (*Fig. S1*). Similarly, studies reporting all types of non-cardiac surgery^2,22,30,33,35,38,43,44,47,49,51,60–62^ had a death rate of 0.23 (95 per cent c.i. 0.07–0.71) per day. These differences were statistically significant at *P* < 0.01.

### Aggregate MACE

At 1-year follow-up, the proportion of patients experiencing MACE events associated with MINS was 0.21^49,56,57,63,64^ (95 per cent c.i. 0.12–0.34), compared with an overall rate of 0.25 (95 per cent c.i. 0.14–0.39), regardless of follow-up interval^28,36,49,53,56,57,63,64^.

**Fig. 5:**
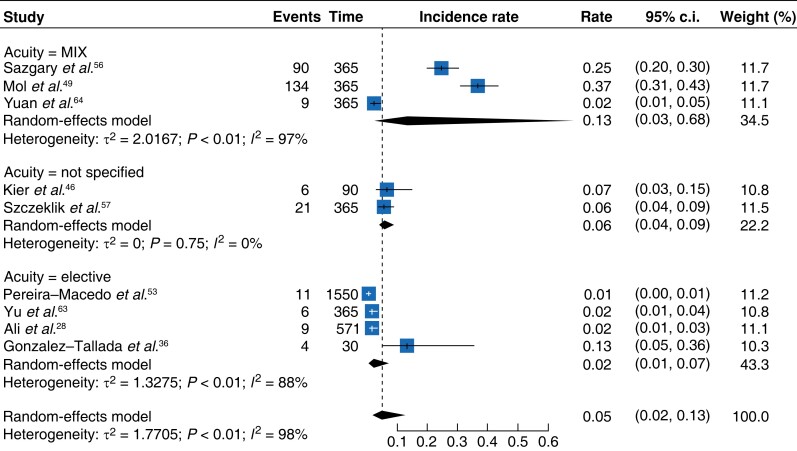
Forest plot showing meta-analysis of cohort studies and MACE rate per day according to acuity

MACE, major adverse cardiac events; MIX, combined emergency and elective.

**Fig. 6:**
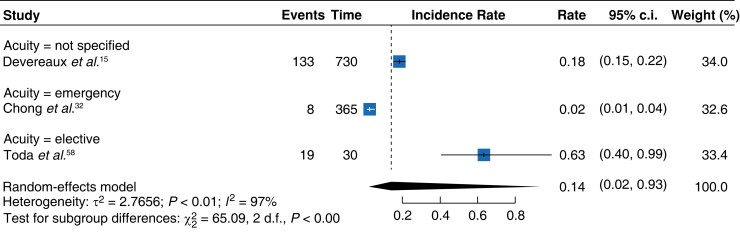
Forest plot showing meta-analysis of control arms and MACE rate per day according to acuity

The meta-analysis of eligible cohort studies showed differing pooled daily MACE rates to control arm studies. A pooled daily rate of 0.05^28,36,46,49,53,56,57,63,64^ (95 per cent c.i. 0.02–0.13) (*Fig. 5*) was demonstrated by the meta-analysis of eligible cohort studies. This was lower in comparison to the control arm studies which showed a MINS-associated MACE daily pooled rate of 0.14^15,32,58^ (95 per cent c.i. 0.02–0.93) (*Fig. 6*).

MACE, major adverse cardiac events.

The MACE rate associated with elective surgery was 0.02 per day (95 per cent c.i. 0.01–0.07)^28,36,53,63^ (*Fig. 5*). There were no available studies reporting MACE outcomes exclusively in emergency surgery; however, studies with mixed acuity demonstrated a MACE rate of 0.13 per day (95 per cent c.i. 0.03–0.68) (*Fig. 5*)^49,56,64^. The difference between these subgroups was statistically significant (*P* < 0.01).

Vascular surgery-specific studies were eligible for meta-analysis which showed an overall MACE rate of 0.02 (95 per cent c.i. 0.01–0.06) per day (*Fig. S2*)^28,53,57^. Studies that had a broader classification of ‘non-cardiac’ surgery demonstrated a pooled MACE rate of 0.20^46,49,56^ (95 per cent c.i. 0.08–0.5). Differences between these two subgroups were statistically significant at *P* < 0.01 (*Fig. S2*).

#### Impact of the use of high-sensitivity troponin

**Fig. 7:**
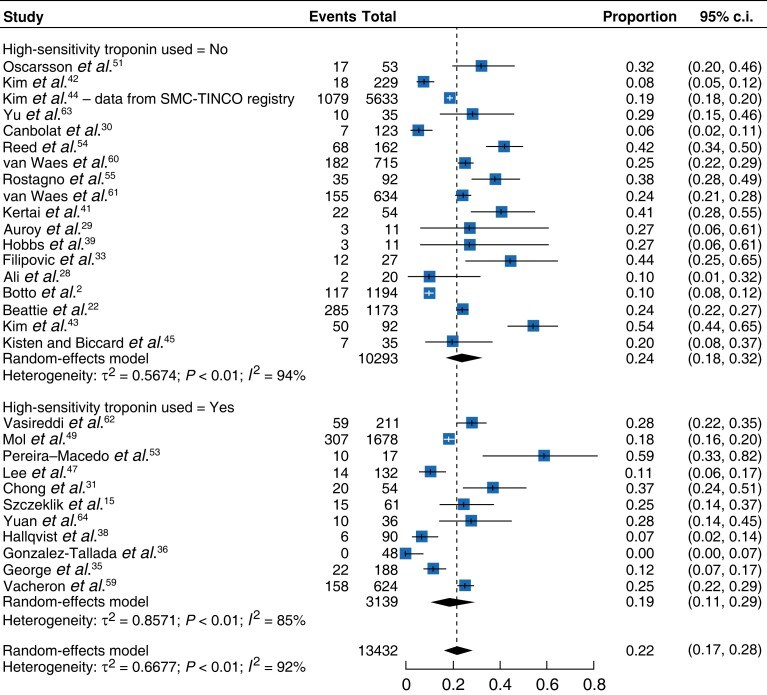
Forest plot showing meta-analysis of death in control arms according to assessment of troponin

The meta-analysis of studies using high-sensitivity troponin^31,32,34–36,38,40,46–49,52,53,56–59,62,64^*versus* other troponins^2,15,22,28–30,33,37,39,41–45,50,51,54,55,60,61,63^ demonstrated pooled mortality rates of 0.19^31,35,36,38,47,49,52,57,59,62,64^ (95 per cent c.i. 0.11–0.29) *versus* 0.24^2,22,28–30,33,39,41–45,51,54,55,60,61,63^ (95 per cent c.i. 0.18–0.32) respectively, which were significantly different (*P* < 0.01, *I*^2^ = 92 per cent) (*Fig. 7*). Similarly, the rate of MACE in the non-high-sensitivity troponin group was 0.28^28,63^ (95 per cent c.i. 0.13–0.51) *versus* 0.20 (95 per cent c.i. 0.10–0.36) for the high-sensitivity troponin group^36,46,49,53,56,57,64^ (*P* < 0.01, *I*^2^ = 96 per cent) (*Fig. S3*).

SMC-TINCO registry, Samsung Medical Center Troponin in Non cardiac Operation registry.

#### Other components of MACE

Further meta-analysis of individual MACE components including myocardial infarction, arrhythmia, heart failure and non-haemorrhagic stroke by follow-up interval is available in *Table 2*. Subgroup analysis by surgical specialty and surgical acuity is detailed in *Tables 3* and *4* respectively. Due to infrequent reporting of arrhythmia and stroke, these outcomes were not suitable for further meta-analyses by subgroups (*Table S1*).

### Bias assessment

Bias assessment revealed a majority of low and moderate bias risk across all six domains. High risk of bias was found in four studies^39,46,60,62^; however, this was confined to study participation in three of the studies^39,46,62^ and attrition in the remaining study^60^. Bias assessments using QUIPS are summarized in *Table S4*.

## Discussion

This systematic review and meta-analysis demonstrates that MINS is associated with both a high mortality rate (25 per cent) and a high rate of MACE (21 per cent) at 1 year after surgery. Notably, this review has highlighted the lack of a standardized diagnostic threshold for MINS as well as inconsistent reporting of MACE and other outcome measures for patients who have developed MINS. The findings of this study have implications in both clinical practice and future research.

It is widely accepted that MINS is associated with an increased 30-day mortality^2^, but there has been limited work exploring outcomes beyond 30 days. A systematic review by Smilowitz *et al*.^18^ found that mortality at 1 year was four times higher in those who had MINS than those who did not (20 *versus* 5.1 per cent). These findings are consistent with the results of this review, although this review goes further and found the mortality rate associated with MINS continues to increase beyond 2 years after surgery and may approach 31 per cent. Notably, this increase is non-linear and many of the events were detected by 1 year. Whilst further longitudinal studies on the long-term sequelae of MINS are required, 1 year may be the plausible biological causation limit of MINS.

The current study explored mortality associated with MINS and the type of surgery performed, as well as the urgency of the procedure. The analysis showed mortality rates associated with MINS varied between different surgical specialties. For example, patients undergoing orthopaedic surgery had an increased mortality rate of 0.30 per day (95 per cent c.i. 0.24–0.37), whilst the mortality rate for vascular surgery patients was 0.26 (95 per cent c.i. 0.15–0.39). Acuity did not appear to impact mortality as subgroup differences were not statistically significant (*P* = 0.14). However, these results may not be representative as it was not possible to differentiate between elective and emergency surgery cases in most included studies with only one study focusing specifically on emergency surgery^31^.

MACE was also highlighted as a major complication following MINS by this review, with an incidence of 21 per cent (95 per cent c.i. 12–34 per cent) at 1 year. A non-linear increase in event rate is also noted between 6 months to 1 year and beyond 2 years. This supports the premise of a standardized postoperative follow-up interval, which will help to improve the fidelity of reporting complications associated with MINS and guide future risk reduction strategies.

The meta-analysis demonstrated that the relationship between surgical acuity and MACE is statistically significant. The elective population had lower rates of MACE events (0.02 per day 95 per cent c.i. 0.01–0.07), compared with a mixed acuity group which included emergency surgeries (0.13 per day 95 per cent c.i. 0.03–0.68) and that this difference was statistically significant (*P* < 0.01). Unfortunately, there was no eligible study available which focused solely on emergency surgery and reported MACE outcomes. Despite this, the results suggest that emergency surgery may have an impact on MACE events post-MINS but further research focusing specifically on emergency surgery is required. This could be possible with clearer reporting and analysis of acuity subgroups in future studies. Improved reporting of types of surgery included in studies would also be beneficial in monitoring outcomes as inadequate numbers of studies were found to allow meta-analysis for MACE components in urology, orthopaedic and thoracic surgery. Whilst the meta-analysis has been carried out for the non-cardiac subgroup, this may have little clinical significance as the subgroup likely contains high heterogeneity within its case mix. Investigating specific risks in subspecialties would be beneficial in understanding different risk profiles for differing types of surgery and may allow improved patient counselling and tailored management strategies in the future.

As this review included studies from 1997 to 2021, a diverse collection of acceptable diagnostic criteria for MINS was observed. For example, inconsistencies in sampling frequency can lead to variations in the incidence of MINS captured by studies and shorter duration of monitoring and troponin sampling could lead to events being missed. Another issue highlighted by Smilowitz *et al.* (2019)^18^ was inappropriately labelling unrelated cardiac events as being precipitated by surgery if the duration of sampling was too long^18^.

Inconsistencies around troponin assays can also lead to inaccurate diagnosis of MINS and therefore outcome reporting. The VISION (Vascular events In non-cardiac Surgery patIents cohOrt evaluatioN) study in 2014 has attempted to delineate the diagnostic criteria of MINS and have suggested a cut-off value for troponin T^2^. The present review has found that this criterion is not universally followed. Interestingly, this meta-analysis has shown that the use of high-sensitivity troponin for the diagnosis of MINS was associated with lower mortality and MACE rates. Potential confounders are that non-high-sensitivity troponin studies may be older with different standards of care to current practice. Alternatively, these results may imply that high-sensitivity troponin is too sensitive and so a higher threshold may be required to reach clinical significance. Notably, heterogeneity within the groups was high and further research specifically focusing on this area would be beneficial.

Furthermore, a wide variety of MACE definitions were also observed from the included studies. This is an important but common issue in cardiovascular research, which has been highlighted by a systematic review in 2021^16^. Inconsistency in definition was also seen within the studies reporting congestive cardiac failure as an outcome measure^2,28,31,32,37,48,53,56,58,64^.

This study is not without limitations. The definition of MINS varies across the literature and reporting of MACE is inconsistent, which probably contributed to the high heterogeneity observed between the included studies. The meta-analysis was limited by the reporting of individual MACE outcomes within published studies which may impact captured event rates. A limited number of studies specifically focusing on defined subgroups, such as surgical acuity and type of surgery, could also potentially lead to increased granularity with comparisons between the subgroups. For example a large proportion of the studies were labelled as ‘not specified’ which did not allow comparisons to be made with other groups. This resulted in only one study focusing on emergency surgery being identified. Similarly, in the surgical type subgroups, only single studies were found to represent thoracic and urological surgery. The meta-analysis of control arms of RCTs was also limited—only three eligible studies were found, which led to high heterogeneity. This high heterogeneity may explain the discordance between the rates demonstrated by meta-analysis in the cohort studies as well as the apparent reduction in MACE and myocardial infarction event rates through time.

The study by Yuan *et al*.^64^ incorrectly used mg/l as a unit of measurement for troponin I which could be due to a printing error. Additionally, none of the studies including congestive cardiac failure as an outcome^2,28,31,32,37,48,53,56,58,64^ differentiated between heart failure with preserved ejection fraction and heart failure with reduced ejection fraction, potentially limiting this analysis.

Despite this, the systematic review and meta-analysis included a wide sample and was conducted in line with PRIMSA and MOOSE guidance^19,21^ and was prospectively registered. These results provide a pragmatic overview of the long-term sequelae of MINS and the range of events associated with it. By only including patients who have a diagnosis, this review was able to specifically focus on MINS-associated outcomes beyond 30 days. By collating the different accepted diagnostic criteria, the findings clearly demonstrate the lack of consistency and standardization in the diagnosis of MINS.

This review has demonstrated significant MACE rates and high long-term mortality associated with MINS. However, the reporting of MACE is inconsistent and the diagnostic criteria for MINS is wide-ranging and lacks uniformity. Future research should aim to establish consistent definitions and sampling frames to diagnose MINS, as well as ensuring key MACE outcomes are reported individually and as an aggregated event count. Researchers should ensure they monitor outcomes to at least 1-year after surgery.

Studies have shown that MINS may be preventable^68^ and it may be possible to mitigate the sequelae of MINS^15,69^. It is imperative that MINS is explored as a modifiable outcome in patients undergoing non-cardiac surgery, particularly in the emergency setting. The initial identification of MINS patients, who are at a higher risk of future MACE, facilitates the development of follow-up as well as secondary prevention strategies. Clinicians might consider whether they wish to routinely assess for MINS in perioperative practice. Recent European Society of Cardiology (ESC) guidelines^70^ recommend routine perioperative troponin screening for at-risk patients undergoing non-cardiac surgery. This highlights the increasing recognition of MINS. Sadly, they do not offer guidance on the management of MINS in this setting, and this represents a major research gap.

## Supplementary Material

zrad021_Supplementary_Data

## Data Availability

All data is included.
