# Supplementary material for: Long-term major adverse cardiovascular events following myocardial injury after non-cardiac surgery: meta-analysis
Source: BJS Open. 2023 Apr 25;7(2):zrad021. doi: 10.1093/bjsopen/zrad021 (PMC10129390; doi:10.1093/bjsopen/zrad021)
Supplement: zrad021_Supplementary_Data [file zrad021_supplementary_data.zip › Supplementary_Material.docx]

Long term major adverse cardiovascular events following myocardial injury after non-cardiac surgery (MINS): meta-analysis

SS Strickland^1^, E Quintela^2,3^, MJ Wilson^2,3^, MJ Lee^1,4^

1 Academic Directorate of General Surgery, Sheffield Teaching Hospitals, Sheffield, UK

2 Department of Anaesthesia, Sheffield Teaching Hospitals, Sheffield, UK

3 Centre for Urgent and Emergency Care Research, School of Health and Related Research, University of Sheffield

4 Department of Oncology and Metabolism, The Medical School, University of Sheffield

**Corresponding author:** Ella Quintela e.quintela@sheffield.ac.uk , School of Health and Related Research, University of Sheffield, UK, ORCID ID: 0000-0001-9367-073X

**Supplementary Materials – Index**

**Supplementary Appendixes**

Appendix S1: Search strategies – Page 2

**Supplementary Figures and Tables**

Figure S1: Forest plot showing meta-analysis of cohort studies: death rates per day by speciality subgroups - Page 6

Figure S2: Forest plot showing meta-analysis of cohort studies: MACE rates per day by specialty subgroups - Page 7

Figure S3: Forest plot showing meta-analysis of cohort studies: MACE events according to troponin assessment used - Page 8

Table S1: Summary of frequency of outcome reporting in studies – Page 9

Table S2: Summary of MINS definitions used by included studies: assays used, cut-off values and sampling frequency – Page 12

Table S3: Summary of Major Adverse Cardiac Events (MACE) definitions by studies which included MACE as an outcome measure – Page 16

Table S4: Summary table of QUIPS bias assessment – Page 17

**Appendix S1**

*Search Strategies*

Medline via OVID

Limits applied: none

1. exp Troponin /

2. exp Troponin I/

3. exp Troponin c/

4. exp Troponin t/

5. exp Creatine Kinase

6. troponin.mp.

7. creatine kinase.mp.

8. myocardial injury.mp.

9. myocardial injuries.mp.

10. myocardial ischemia.mp.

11. MINS.ti.

12. 1 or 2 or 3 or 4 or 5 or 6 or 7 or 8 or 9 or 10 or 11

13. Noncardiac surgery.mp.

14. Noncardiac surgeries.mp.

15. Non-cardiac surgery.mp.

16. Non-cardiac surgeries.mp.

17. Non-cardiac surgical.mp.

18. Noncardiac surgical.mp.

19. Vascular surgery.mp.

20. Vascular surgeries.mp.

21. Vascular surgical.mp.

22. Vascular procedure*.mp.

23. Orthop?edic surgery.mp.

24. Orthop?edic surgeries.mp.

25. Orthop?edic surgical.mp.

26. Orthop?edic procedure*.mp.

27. Exp Orthopedic Procedures/

28. Exp vascular surgical procedures/

29. 10 or 11 or 12 or 13 or 14 or 15 or 16 or 17 or 18 or 19 or 20 or 21 or 22 or 23 or 24 or 25 or 26 or 27 or 28

30. Exp perioperative period/

31. Exp postoperative period/

32. Exp postoperative care/

33. Exp perioperative care/

34. Perioperative.mp.

35. Peri-operative.mp.

36. Postoperative.mp.

37. Post-operative.mp.

38. 30 or 31 or 32 or 33 or 34 or 35 or 36 or 37

39. 12 and 29 and 38

Database Used: EMBASE via Ovid

Limits Applied: None

1974-2021

1. troponin/

2. troponin t/

3. troponin i/

4. troponin c/

5. creatine kinase/

6. "troponin".mp.

7. "creatine kinase".mp.

8. "myocardial injury".mp.

9. "myocardial injuries".mp.

10. “myocardial ischemia”.mp

11. "MINS".ti.

12. 1 or 2 or 3 or 4 or 5 or 6 or 7 or 8 or 9 or 10 or 11

13. "noncardiac surgery".mp.

14. "noncardiac surgeries".mp.

15. "non-cardiac surgery".mp.

16. "non-cardiac surgeries".mp.

17. "non-cardiac surgical".mp.

18. "noncardiac surgical".mp.

19. "vascular surgery ".mp.

20. "vascular surgeries".mp.

21. "vascular surgical".mp.

22. "vascular procedure*".mp.

23. "orthop?edic surgery".mp.

24. "orthop?edic surgeries".mp.

25. "orthop?edic surgical".mp.

26. "orthop?edic procedure*".mp.

27. exp orthopedic procedures/

28. exp vascular surgical procedures/

29. 13 or 14 or 15 or 16 or 17 or 18 or 19 or 20 or 21 or 22 or 23 or 24 or 25 or 26 or 27 or 28.

30. exp perioperative period/

31. Exp postoperative period/

32. Exp postoperative care/

33. Exp perioperative care/

34. "perioperative".mp.

35. "peri-operative".mp.

36. "postoperative".mp.

37. "post-operative".mp.

38. 30 or 31 or 32 or 33 or 34 or 35 or 36 or 36 or 37

39. 12 and 29 and 38

Database Used: Cochrane CENTRAL

Limits Applied: None

1. Troponin MESH exp all trees

2. Troponin t MESH exp all trees

3. Troponin i MESH exp all trees

4. Troponin c MESH exp all trees

5. Creatine kinase MESH exp all trees

6. (Troponin): ti,ab,kw

7. (creatine kinase):ti,ab,kw .

8. (myocardial injury):ti,ab,kw

9. (myocardial injuries):ti,ab,kw

10. (myocardial ischemia): ti,ab,kw

11. (MINS):ti.

12. #1 or #2 or #3 or #4 or #5 or #6 or #7 or #8 or #9 or #10 or #11

13. (noncardiac surgery): ti,ab,kw

14. (noncardiac surgeries): ti,ab,kw

15. (non-cardiac surgery): ti,ab,kw .

16. (non-cardiac surgeries): ti,ab,kw

17. (non-cardiac surgical): ti,ab,kw

18. (noncardiac surgical): ti,ab,kw

19. (vascular surgery): ti,ab,kw

20. (vascular surgeries): ti,ab,kw

21. (vascular surgical): ti,ab,kw

22. (vascular procedure*): ti,ab,kw

23. (orthop?edic surgery): ti,ab,kw

24. (orthop?edic surgeries): ti,ab,kw

25. (orthop?edic surgical): ti,ab,kw

26. (orthop?edic procedure*): ti,ab,kw .

27. orthopedic procedures MESH exp all trees

28. vascular surgical procedures MESH exp all trees

29. #13 or #14 or #15 or #16 or #17 or #18 or #19 or #20 or #21 or #22 or #23 or #24 or #25 or #26 or #27 or #28.

30. perioperative period MESH exp all tress

31. postoperative period MESH exp all trees

32. postoperative care MESH exp all trees

33. perioperative care MESH exp all trees

34. (Perioperative): ti,ab,kw

35. (Peri-operative): ti,ab,kw

36. (Postoperative): ti,ab,kw

37. (Post-operative): ti,ab,kw

38. #30 or #31 or #32 or #33 or #34 or #35 or #36 or #37

39. #12 and #29 and #38

Figure S1: Forest plot showing meta-analysis of cohort studies: death rates per day by speciality subgroups


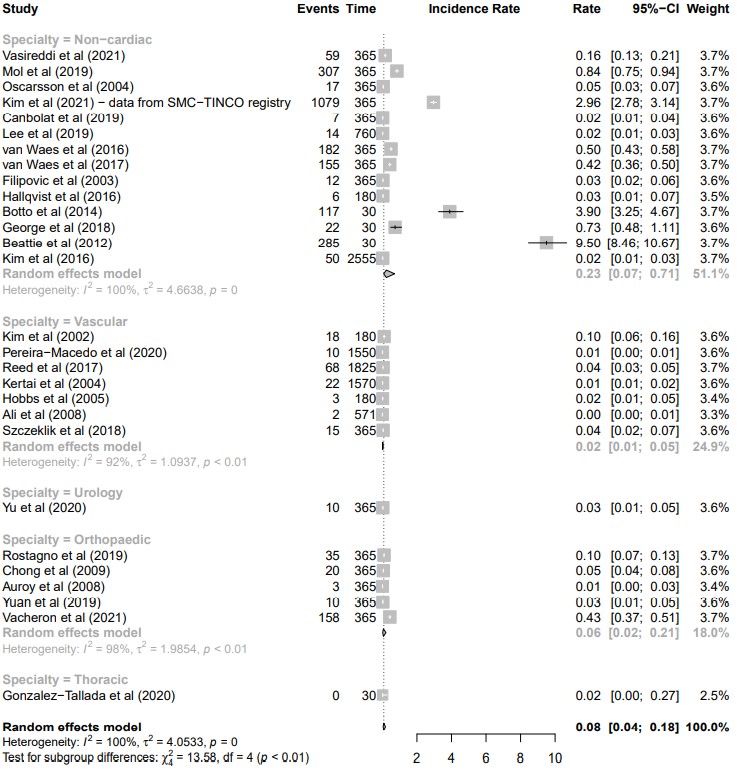


Figure S2: Forest plot showing meta-analysis of cohort studies: MACE rates per day by specialty subgroups

^
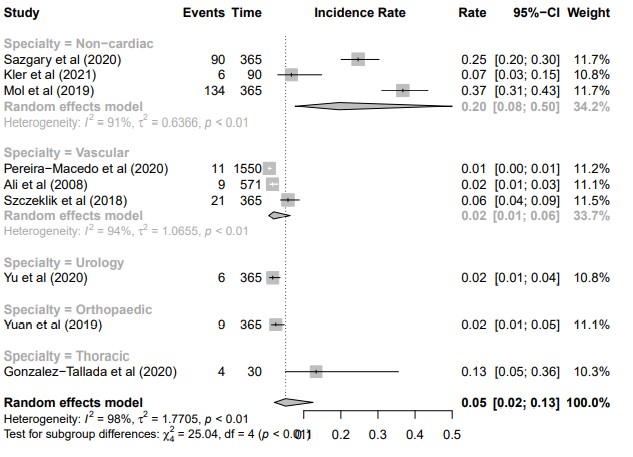
^

Figure S3: Forest plot showing meta-analysis of cohort studies: MACE events according to troponin assessment used -


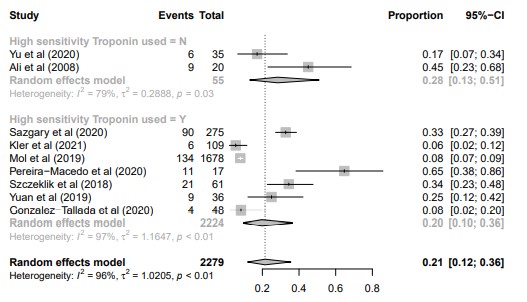


*Table S1: Summary of Frequency of Outcome Reporting in studies*

| **Author (year)** | **MI** | **Non-hemorrhagic stroke** | **Arrhythmia** | **Heart failure** | **Peripheral arterial thrombus** | **Cardiac arrest** | **Amputation** | **Death or Mortality** | **MACE** |
| --- | --- | --- | --- | --- | --- | --- | --- | --- | --- |
| ***Ali et al 2008*** [*^47^*](https://paperpile.com/c/QsyolI/AO4yd) | X |  |  | X |  |  |  | X | X |
| ***Auroy et al 2008*** [*^55^*](https://paperpile.com/c/QsyolI/oWH1m) | X |  |  |  |  |  |  | X |  |
| ***Beattie et al 2012*** [*^26^*](https://paperpile.com/c/QsyolI/xUJ2M) |  |  |  |  |  |  |  | X |  |
| ***Botto et al 2014*** [*^2^*](https://paperpile.com/c/QsyolI/kn20Q) |  | X |  | X |  | X |  | X | X |
| ***Canbolat et al 2014*** [*^27^*](https://paperpile.com/c/QsyolI/CohHJ) |  |  |  |  |  |  |  | X |  |
| ***Chong et al 2009*** [*^56^*](https://paperpile.com/c/QsyolI/u0bJM) | X |  | X | X |  |  |  | X |  |
| ***Chong et al 2012*** [*^57^*](https://paperpile.com/c/QsyolI/MnQds)  **CA RCT** | X |  | X | X |  |  |  | X | X |
| ***Devereaux et al 2018*** [*^15^*](https://paperpile.com/c/QsyolI/H2hbV) **CA RCT** | X | X |  |  | X |  |  | X | X |
| ***Filipovic et al 2003*** [*^28^*](https://paperpile.com/c/QsyolI/hSXw3) |  |  |  |  |  |  |  | X |  |
| ***Genc Moralar et al 2021*** [*^46^*](https://paperpile.com/c/QsyolI/4ITGX) |  |  |  |  |  |  |  | X |  |
| ***George et al 2018*** [*^29^*](https://paperpile.com/c/QsyolI/mZM9O) |  |  |  |  |  |  |  | X |  |
| ***Gonzalez- Tallada et al 2020*** [*^63^*](https://paperpile.com/c/QsyolI/9HiwS) |  |  | X |  |  |  |  | X | X |
| ***Gouda et al 2021*** [*^30^*](https://paperpile.com/c/QsyolI/tuH11) | X |  |  | X |  |  |  | X |  |
| ***Hallqvist et al 2016*** [*^31^*](https://paperpile.com/c/QsyolI/9LA4J) |  |  |  |  |  |  |  | X |  |
| ***Hobbs et al 2005*** [*^48^*](https://paperpile.com/c/QsyolI/vPOPY) |  |  |  |  |  |  |  | X |  |
| ***Jackson et al 2018*** [*^32^*](https://paperpile.com/c/QsyolI/fZ9iU) |  |  |  |  |  |  |  | X |  |
| ***Kertai et al 2004*** [*^49^*](https://paperpile.com/c/QsyolI/6lzEp) |  |  |  |  |  |  |  | X |  |
| ***Kim et al 2002*** [*^50^*](https://paperpile.com/c/QsyolI/T7jjt) |  |  |  |  |  |  |  | X |  |
| ***Kim et al 2016*** [*^33^*](https://paperpile.com/c/QsyolI/K59VK) |  |  |  |  |  |  |  | X |  |
| ***Kim et al 2021*** [*^34^*](https://paperpile.com/c/QsyolI/XI8v4) |  |  |  |  |  |  |  | X |  |
| ***Kisten and Biccard 2016*** [*^51^*](https://paperpile.com/c/QsyolI/2vNE6) |  |  |  |  |  |  |  | X |  |
| ***Kler et al 2021*** [*^35^*](https://paperpile.com/c/QsyolI/fZPVx) |  |  |  |  |  |  |  | X | X |
| ***Lee et al 2019*** [*^36^*](https://paperpile.com/c/QsyolI/xo9Qs) |  |  |  |  |  |  |  | X |  |
| ***Lee et al 2020*** [*^37^*](https://paperpile.com/c/QsyolI/xWA0g) | X |  | X | X |  |  |  | X |  |
| ***Mol et al 2019*** [*^39^*](https://paperpile.com/c/QsyolI/8u31l) |  |  |  |  |  |  |  | X | X |
| ***Oberweis et al 2015*** [*^58^*](https://paperpile.com/c/QsyolI/N9Dy6) | X |  |  |  |  |  |  |  |  |
| ***Oscarsson et al 2004*** [*^40^*](https://paperpile.com/c/QsyolI/pIGA9) | X |  |  |  |  |  |  | X |  |
| ***Pereira-Macedo et al 2019*** [*^52^*](https://paperpile.com/c/QsyolI/dyVyr) | X | X |  |  |  |  |  | X | X |
| ***Pereira-Macedo et al 2020*** [*^53^*](https://paperpile.com/c/QsyolI/7w8Ax) | X |  |  | X | X |  |  | X | X |
| ***Reed et al 2017*** [*^54^*](https://paperpile.com/c/QsyolI/5H6cO) |  |  |  |  |  |  |  | X |  |
| ***Rostagno et al 2019*** [*^59^*](https://paperpile.com/c/QsyolI/PugIJ) |  | X |  |  |  | X |  | X |  |
| ***Sazgary et al 2020*** [*^41^*](https://paperpile.com/c/QsyolI/kQbIL) | X |  | X | X |  |  |  |  | X |
| ***Szczeklik et al 2018*** [*^65^*](https://paperpile.com/c/QsyolI/yfvMz) | X | X |  |  |  |  | X | X | X |
| ***Toda et al 2020*** [*^42^*](https://paperpile.com/c/QsyolI/TeDQS)  **CA RCT** | X | X | X | X |  |  |  | X | X |
| ***Vacheron et al 2021*** [*^60^*](https://paperpile.com/c/QsyolI/XhRTp) | X |  |  |  |  |  |  | X |  |
| ***Van Waes et al 2016*** [*^43^*](https://paperpile.com/c/QsyolI/V2iWg) | X |  |  |  |  |  |  | X |  |
| ***Van Waes et al 2017*** [*^44^*](https://paperpile.com/c/QsyolI/lQZp6) | X |  |  |  |  |  |  | X |  |
| ***Vasireddi et al 2021*** [*^45^*](https://paperpile.com/c/QsyolI/TN6hV) |  |  |  |  |  |  |  | X |  |
| ***Yu et al 2020*** [*^62^*](https://paperpile.com/c/QsyolI/NBUEK) |  |  |  |  |  |  |  | X | X |
| ***Yuan et al 2019*** [*^61^*](https://paperpile.com/c/QsyolI/iEdiz) | X |  |  | X |  |  |  | X | X |

MI = Myocardial Infarction; MACE = Major Adverse Cardiac Events; CA RCT = Control Arm of Randomised Control Trial

*Table S2: Summary of MINS definitions used by included studies: assays used, cut-off values and sampling frequency*

| **Study** | **Troponin assay** | **Cut-off value indicative of MINS** | **Sampling frequency** |
| --- | --- | --- | --- |
| **Ali et al (2008)** [^47^](https://paperpile.com/c/QsyolI/AO4yd) | Troponin I (Dade Behring) | Peak serum level >0.54ng/mL | Days 1, 3 and 7 post surgery |
| **Auroy et al (2008)** [^55^](https://paperpile.com/c/QsyolI/oWH1m) | Cardiac troponin I (Ortho Vitros ECi; Ortho-Clinical Diagnostics) | >0.08ng/mL (values above 99th percentile) | On the morning of the first three days post surgery |
| **Beattie et al (2012)** [^26^](https://paperpile.com/c/QsyolI/xUJ2M) | Troponin I (Siemens) | Peak postoperative level >0.07mcg/ml | Any time within 30 days of surgery |
| **Botto et al (2014)** [^2^](https://paperpile.com/c/QsyolI/kn20Q) | Fourth generation troponin T | 0.04ng/ml | 6-12 hours postoperatively as well as day 1, 2 and 3 post surgery |
| **Canbolat et al (2019)**[^27^](https://paperpile.com/c/QsyolI/CohHJ) | Cardiac Troponin I (Assay not specified) | 0.04ng/ml | Every 24 hours in the first 3 days post surgery |
| **Chong et al (2009)**[^56^](https://paperpile.com/c/QsyolI/u0bJM) | Troponin I (Architect STAT, Abbott) | Troponin rise of >0.03mcg/l in postop trop vs pre-op level | Pre-operatively and days 1-3 post surgery. If a patient had less than 3 post op troponin samples, further samples taken at days 4-7 |
| **Chong et al (2012)** [^57^](https://paperpile.com/c/QsyolI/MnQds) | Troponin I (Beckmann Coulter) | >0.05 microgram/L | Pre-operatively and days 1-3 post surgery |
| **Devereaux et al (2018)** [^15^](https://paperpile.com/c/QsyolI/H2hbV) | Assay not specified | Elevated troponin (cut offs not specified) | Obtain samples for at least the first 2 days after surgery |
| **Filipovic et al (2003)** [^28^](https://paperpile.com/c/QsyolI/hSXw3) | Troponin I (Abbott) | >2mcg/L | Before surgery and immediately after arrival in recovery, 8 hours after the end of surgery and daily for the first 3 days post surgery and on day 6 after surgery |
| **Genc Moralar et al (2021)** [^46^](https://paperpile.com/c/QsyolI/4ITGX) | High sensitivity troponin T (Elecsys 2010, Roche) | Peak >0.03ng/ml | First 3 days post surgery |
| **George et al (2018)** [^29^](https://paperpile.com/c/QsyolI/mZM9O) | High sensitivity troponin I (Abbott) | Peak >0.03ng/dL | 12 and 24 hours following surgery and further sampling at 48 hours if initial levels qualify for MINS |
| **Gonzalez-Tallada et al (2020)** [^63^](https://paperpile.com/c/QsyolI/9HiwS) | High sensitivity troponin I (Centaur TnI-Ultra, Siemens) | Peak >0.04ng/ml | Day 1 and 2 post surgery |
| **Gouda et al (2021)** [^30^](https://paperpile.com/c/QsyolI/tuH11) | Any troponin assay | > Upper limit of normal | Not specified |
| **Hallqvist et al (2016)** [^31^](https://paperpile.com/c/QsyolI/9LA4J) | High sensitivity cTnT (Elecsys 2010, Roche Diagnostics) | >14ng/L | First postoperative morning |
| **Hobbs et al (2005)** [^48^](https://paperpile.com/c/QsyolI/vPOPY) | Cardiac Troponin I (OPUS, Dade Behring) | >0.5ng/ml in any of the four samples | 6 hours post surgery, and on mornings of postoperative days 1, 2 and 3 |
| **Jackson et al (2018)** [^32^](https://paperpile.com/c/QsyolI/fZ9iU) | Troponin I (Adiva Centaur, Siemens) | >17ng/L | Measured on any samples taken within the first 72 hours post surgery |
| **Kertai et al (2004)** [^49^](https://paperpile.com/c/QsyolI/6lzEp) | Cardiac troponin T (TropT version 2, Roche Diagnostics) | >0.1ng/ml | Days 2, 3 and 7 post surgery or at discharge |
| **Kim et al (2002)** [^50^](https://paperpile.com/c/QsyolI/T7jjt) | Cardiac troponin I (Stratus fluorometric enzyme immunoassay (Dade Pharmaceuticals)) | Peak >1.5ng/ml | Immediately after surgery, morning of days 1, 2 and 3 post surgery |
| **Kim et al (2016)** [^33^](https://paperpile.com/c/QsyolI/K59VK) | Troponin I (Abbott) | >0.1ng/ml | Day 1 and 2 post surgery |
| **Kim et al (2021)** [^34^](https://paperpile.com/c/QsyolI/XI8v4) | Cardiac troponin I (Advia Centaur XP, Siemens Healthcare Diagnostics) | ≥0.04 ng/ml | Within 30 days after surgery |
| **Kisten and Biccard (2016)** [^51^](https://paperpile.com/c/QsyolI/2vNE6) | Troponin I (Advia Centaur XP, Siemens) | >40ng/l but < 600ng/l | Day of operation and during first 3 days post surgery |
| **Kler et al (2021)** [^35^](https://paperpile.com/c/QsyolI/fZPVx) | Fifth generation hs-TnT | >21ng/l (derived from ROC curves in study) | Days 1, 2 and 3 post surgery |
| **Lee et al (2019)** [^36^](https://paperpile.com/c/QsyolI/xo9Qs) | High sensitivity cardiac troponin T (Advia Centaur XR, Siemens) | >0.04ng/ml | Within first 2 days post surgery |
| **Lee et al (2020)** [^37^](https://paperpile.com/c/QsyolI/xWA0g) | High sensitivity cardiac troponin I (Advia Centaur XP, Siemens) | Above the 99th percentile of upper reference limit based on the 4th universal definition of myocardial infarction | 48 hours before and after surgery |
| **Mol et al (2019)** [^39^](https://paperpile.com/c/QsyolI/8u31l) | Fifth generation high sensitivity troponin T (Elecsys Roche diagnostics) | > 14ng/l (99th % limit of normal) | Day 1-3 post surgery |
| **Oberweis et al (2015)** [^58^](https://paperpile.com/c/QsyolI/N9Dy6) | Cardiac troponin I  (VITROS cTnI ES assay) ot ST AIA-PACK second generation cTnI assay | Elevation greater than the 99th percentile upper reference limit of assays  (0.04 and 0.06 ng/ml, respectively) | Not specified |
| **Oscarsson et al (2004)** [^40^](https://paperpile.com/c/QsyolI/pIGA9) | Troponin T (Elecsys 2010 - Roche Diagnostics) | >0.02ng/ml | Baseline (1 hour pre-induction of anaesthesia) and within day 5-7 post surgery |
| **Pereira-Macedo et al (2019)** [^52^](https://paperpile.com/c/QsyolI/dyVyr) | Trop I immunoassay (Architect Stat Troponin I, Abbot Laboratories) and  a fourth-generation assay hSTnI (Abbot Laboratories) | 0.032 μg/mL cTn1 regardless of sex and 27 ng/mL hsTnI (male) or 11.4 ng/ mL (female) | Within first 48 hours post surgery |
| **Pereira-Macedo et al (2020)** [^53^](https://paperpile.com/c/QsyolI/7w8Ax) | Cardiac Troponin I (Architect Stat Troponin I, Abbott), High sensitivity troponin I (Abbott) | Elevation above the 99th percentile reference limit: >0.32mcg/ml for cTnI, for hsTnI 11.4ng/ml (female) and 27ng/ml (male) | Immediately after surgery and at day 1-2 post surgery |
| **Reed et al (2017)** [^54^](https://paperpile.com/c/QsyolI/5H6cO) | Cardiac troponin T (Roche 4th generation) | <0.01ng/ml - not sampled, 0.01-0.29ng/ml, 0.03-0.099ng/ml, >0.4ng/ml | Within 96 hours of surgery |
| **Rostagno et al (2019)** [^59^](https://paperpile.com/c/QsyolI/PugIJ) | Troponin assay not specified | Perioperative troponin increase or peak value of >0.5mcg/l | At the end of surgery and at 12, 24 and 48 hours post surgery |
| **Sazgary et al (2020)** [^41^](https://paperpile.com/c/QsyolI/kQbIL) | High sensitivity cardiac troponin T - Elecsys (Roche Diagnostics) | Absolute increase >14ng/L above pre-op levels or between 2 postoperative levels if no pre-op measurements available | Pre-operatively and at day 1 and 2 post surgery |
| **Szczeklik et al (2018)** [^65^](https://paperpile.com/c/QsyolI/yfvMz) | High sensitivity troponin T (Elecsys 2010 Roche) | Peak value >14ng/L with a relative increase >30% from the baseline hsTnT value | Admission, 3-6 hours following procedure and at the following morning |
| **Toda et al (2020)** [^42^](https://paperpile.com/c/QsyolI/TeDQS) | Fifth generation high sensitivity cardiac troponin T (Roche) | >0.014ng/ml and relative hs-cTnT change >20% | Pre-operatively and on post operative days 1 and 3 |
| **Vacheron et al (2021)** [^60^](https://paperpile.com/c/QsyolI/XhRTp) | High sensitivity troponin I (Architect stat, Abbott) | >5ng/L | Days 1, 2 and 3 post surgery |
| **van Waes et al (2016)** [^43^](https://paperpile.com/c/QsyolI/V2iWg) | Troponin I (AccuTnI assay, Beckman) | >0.06mcg/L (10% coefficient of variation above the 99th percentile) | Daily in the first 3 days after surgery |
| **van Waes et al (2017)** [^44^](https://paperpile.com/c/QsyolI/lQZp6) | Troponin I (assay not specified) | >60ng/L | Daily in the first 3 days after surgery |
| **Vasireddi et al (2021)** [^45^](https://paperpile.com/c/QsyolI/TN6hV) | Troponin I (Beckman Coulter Access AccuTnI+3) | >0.06ng/ml (x2 above the upper limit of normal which was 0.03ng/ml) | Within 14 days post surgery |
| **Yu et al (2020)** [^62^](https://paperpile.com/c/QsyolI/NBUEK) | Troponin I (assay not specified) | >0.04mcg/L | Within 3 days post surgery |
| **Yuan et al (2018)** [^61^](https://paperpile.com/c/QsyolI/iEdiz) | Troponin I (Johnson Vitros ECiQ) | >0.03mg/L* | Next day of admission, day 1 and 5 post surgery |
| *Potential printing error | | | |

*Table S3:* Summary of Major Adverse Cardiac Events (MACE) definitions by studies which included MACE as an outcome measure

| **Study** | **Composite MACE definition** |
| --- | --- |
| Ali et al 2008 | Occurrence of death, congestive cardiac failure, or myocardial infarction |
| Botto et al 2014 | Composite of mortality, nonfatal cardiac arrest, nonfatal congestive cardiac failure, and nonfatal stroke |
| Chong et al 2012 (Ca RCT) | Does not specify MACE components |
| Deveraux et al 2018 (CA RCT) | Composite of vascular mortality and non-fatal myocardial infarction, non-haemorrhagic stroke, peripheral arterial thrombosis, amputation, and symptomatic venous thromboembolism |
| Gonzalez-Tallada et al 2020 (CA RCT) | Does not specify MACE components |
| Kier et al 2021 | Does not specify MACE components |
| Mol et al 2019 | Occurrence of myocardial infarction, angina, revascularization therapy or cerebrovascular accident in the first year after surgery |
| Pereira-Macedo et al 2019 | Composite of MI, AHF and all-cause mortality |
| Pereira-Macedo et al 2020 | Composite outcome of MI, acute heart failure (AHF), and all-cause mortality |
| Sazgary et al 2020 | Includes cardiovascular death (CVD), acute heart failure (AHF), haemodynamically relevant arrhythmias, spontaneous myocardial infarction (MI), and perioperative myocardial infarction/injury (PMI) |
| Szczeklik et al 2018 | Composite of myocardial infarction, stroke, and death (by any cause) |
| Toda et al 2020 | Non-fatal myocardial infarction and stroke, decompensated heart failure and tachyarrhythmia including new-onset atrial fibrillation (AF), atrial tachycardia, paroxysmal supraventricular tachycardia, inappropriate sinus tachycardia and ventricular tachycardia ≤30 days after surgery |
| Yu et al 2020 | One or more of the following complications within 6 months: arrhythmia, nonfatal cardiac arrest, acute myocardial infarction, heart failure, or cerebrovascular accident. |
| Yuan et al 2019 | Cardiac events included readmission owing to worsening heart failure, or arrhythmia, or intervention. |

*Table S4: Summary Table of QUIPS Bias Assessments*

|  | **Study Participation** | **Study Attrition** | **Prognostic Factor** | **Outcome Measure** | **Confounding** | **Statistics** |
| --- | --- | --- | --- | --- | --- | --- |
| ***Ali et al 2008*** [*^47^*](https://paperpile.com/c/QsyolI/AO4yd) | M | L | M | M | L | M |
| ***Auroy et al 2008*** [*^55^*](https://paperpile.com/c/QsyolI/oWH1m) | M | M | M | M | M | M |
| ***Beattie et al 2012*** [*^26^*](https://paperpile.com/c/QsyolI/xUJ2M) | M | L | M | L | M | L |
| ***Botto et al 2014*** [*^2^*](https://paperpile.com/c/QsyolI/kn20Q) | L | L | L | L | L | L |
| ***Canbolat et al 2014*** [*^27^*](https://paperpile.com/c/QsyolI/CohHJ) | M | L | M | L | M | L |
| ***Chong et al 2009*** [*^56^*](https://paperpile.com/c/QsyolI/u0bJM) | M | L | M | L | L | L |
| ***Chong et al 2012*** [*^57^*](https://paperpile.com/c/QsyolI/MnQds) | M | L | M | L | L | L |
| ***Devereaux et al 2018*** [*^15^*](https://paperpile.com/c/QsyolI/H2hbV) | L | M | L | L | L | L |
| ***Filipovic et al 2003*** [*^28^*](https://paperpile.com/c/QsyolI/hSXw3) | M | L | M | M | M | L |
| ***Genc Moralar et al 2021*** [*^46^*](https://paperpile.com/c/QsyolI/4ITGX) | M | L | M | L | M | M |
| ***George et al 2018*** [*^29^*](https://paperpile.com/c/QsyolI/mZM9O) | M | L | M | L | L | L |
| ***Gonzalez- Tallada et al 2020*** [*^63^*](https://paperpile.com/c/QsyolI/9HiwS) | M | M | M | L | M | L |
| ***Gouda et al 2021*** [*^30^*](https://paperpile.com/c/QsyolI/tuH11) | M | L | L | L | M | L |
| ***Hallqvist et al 2016*** [*^31^*](https://paperpile.com/c/QsyolI/9LA4J) | M | M | M | L | M | L |
| ***Hobbs et al 2005*** [*^48^*](https://paperpile.com/c/QsyolI/vPOPY) | H | M | M | M | M | M |
| ***Jackson et al 2018*** [*^32^*](https://paperpile.com/c/QsyolI/fZ9iU) | M | M | M | L | L | L |
| ***Kertai et al 2004*** [*^49^*](https://paperpile.com/c/QsyolI/6lzEp) | M | L | L | M | M | L |
| ***Kim et al 2002*** [*^50^*](https://paperpile.com/c/QsyolI/T7jjt) | M | M | L | M | M | L |
| ***Kim et al 2016*** [*^33^*](https://paperpile.com/c/QsyolI/K59VK) | M | L | M | M | M | L |
| ***Kim et al 2021*** [*^34^*](https://paperpile.com/c/QsyolI/XI8v4) | M | L | M | L | M | L |
| ***Kisten and Biccard 2016*** [*^51^*](https://paperpile.com/c/QsyolI/2vNE6) | M | L | L | L | M | M |
| ***Kler et al 2021*** [*^35^*](https://paperpile.com/c/QsyolI/fZPVx) | H | L | M | L | L | L |
| ***Lee et al 2019*** [*^36^*](https://paperpile.com/c/QsyolI/xo9Qs) | **M** | **L** | **M** | **M** | **M** | **L** |
| ***Lee et al 2020*** [*^37^*](https://paperpile.com/c/QsyolI/xWA0g) | **M** | **L** | **L** | **M** | **M** | **M** |
| ***Mol et al 2019*** [*^39^*](https://paperpile.com/c/QsyolI/8u31l) | M | M | M | L | M | L |
| ***Oberweis et al 2015*** [*^58^*](https://paperpile.com/c/QsyolI/N9Dy6) | M | L | M | L | M | L |
| ***Oscarsson et al 2004*** [*^40^*](https://paperpile.com/c/QsyolI/pIGA9) | L | M | M | M | M | L |
| ***Pereira-Macedo et al 2019*** [*^52^*](https://paperpile.com/c/QsyolI/dyVyr) | L | L | L | L | M | L |
| ***Pereira-Macedo et al 2020*** [*^53^*](https://paperpile.com/c/QsyolI/7w8Ax) | M | M | M | L | M | L |
| ***Reed et al 2017*** [*^54^*](https://paperpile.com/c/QsyolI/5H6cO) | M | L | M | L | L | L |
| ***Rostagno et al 2019*** [*^59^*](https://paperpile.com/c/QsyolI/PugIJ) | M | M | M | M | M | L |
| ***Sazgary et al 2020*** [*^41^*](https://paperpile.com/c/QsyolI/kQbIL) | M | M | M | L | M | L |
| ***Szczeklik et al 2018*** [*^65^*](https://paperpile.com/c/QsyolI/yfvMz) | M | L | M | L | M | M |
| ***Toda et al 2020*** [*^42^*](https://paperpile.com/c/QsyolI/TeDQS) | M | L | L | M | M | M |
| ***Van Waes et al 2016*** [*^43^*](https://paperpile.com/c/QsyolI/V2iWg) | M | H | M | L | M | L |
| ***Van Waes et al 2017*** [*^44^*](https://paperpile.com/c/QsyolI/lQZp6) | M | L | L | L | M | L |
| ***Vacheron et al 2021***[*^60^*](https://paperpile.com/c/QsyolI/XhRTp) | M | L | M | M | M | M |
| ***Vasireddi et al 2021*** [*^45^*](https://paperpile.com/c/QsyolI/TN6hV) | H | L | L | L | L | L |
| ***Yu et al 2020*** [*^62^*](https://paperpile.com/c/QsyolI/NBUEK) | M | L | M | L | M | L |
| ***Yuan et al 2019*** [*^61^*](https://paperpile.com/c/QsyolI/iEdiz) | M | L | M | L | L | L |

H = high risk of bias; M = medium risk of bias; L = low risk of bias
